# Effect of Rare Earth Ce on Deep Stamping Properties of High-Strength Interstitial-Free Steel Containing Phosphorus

**DOI:** 10.3390/ma13061473

**Published:** 2020-03-24

**Authors:** Hao Wang, Yanping Bao, Chengyi Duan, Lu Lu, Yan Liu, Qi Zhang

**Affiliations:** 1 State Key Laboratory of Advanced Metallurgy, University of Science and Technology Beijing, Beijing 100083, China; wanghao_bisg@163.com; 2 Technical Center of Inner Mongolia Baotou Steel Union Co., Ltd., Baotou 014010, China; duancy73@126.com (C.D.); jszzgsll@163.com (L.L.); victory_523@126.com (Q.Z.); 3 Inner Mongolia Enterprise Key Laboratory of Rare Earth Steel Products Research & Development, Baotou 014010, China

**Keywords:** high-strength IF steel containing P, Fe(Nb + Ti)P phase, rare earth Ce, deep stamping properties

## Abstract

The influence of rare earth Ce on the deep stamping property of high-strength interstitial-free (IF) steel containing phosphorus was analyzed. After adding 120 kg ferrocerium alloy (Ce content is 10%) in the steel, the inclusion statistics and the two-dimensional morphology of the samples in the direction of 1/4 thickness of slab and each rolling process were observed and compared by scanning electron microscope (SEM). After the samples in each rolling process were treated by acid leaching, the three-dimensional morphology and components of the second phase precipitates were observed by SEM and energy dispersive spectrometer (EDS). The microstructure of the sample was observed by optical microscope, and the grain size was compared. Meanwhile, the content and strength of the favorable texture were analyzed by X-ray diffraction (XRD). Finally, the mechanical properties of the product were analyzed. The results showed that: (1) The combination of rare earth Ce with activity O and S in steel had lower Gibbs free energy, and it was easy to generate CeAlO_3_, Ce_2_O_2_S, and Ce_2_O_3_. The inclusions size was obviously reduced, but the number of inclusions was increased after adding rare earth. The morphology of inclusions changed from chain and strip to spherical. The size of rare earth inclusions was mostly about 2–5 μm, distributed and dispersed, and their elastic modulus was close to that of steel matrix, which was conducive to improving the structure continuity of steel. (2) The rare earth compound had a high melting point. As a heterogeneous nucleation point, the nucleation rate was increased and the solidification structure was refined. The grade of grain size of products was increased by 1.5 grades, which is helpful to improve the strength and plasticity of metal. (3) Rare earth Ce can inhibit the segregation of P element at the grain boundary and the precipitation of Fe(Nb+Ti)P phase. It can effectively increase the solid solution amount of P element in steel, improve the solid solution strengthening effect of P element in high-strength IF steel, and obtain a large proportion of {111} favorable texture, which is conducive to improving the stamping formability index r_90_ value.

## 1. Introduction

Aluminum deoxidized interstitial-free (IF) steel has become the main material for automobile outer panel production in the world because of its good deep stamping performance and economy [1,2]. With the development of the automobile to the direction of weight reduction and lighter weight, the automobile plate with high-strength and ultra-deep stamping performance has been developed rapidly [3,4]. The solid solution strengthened IF steel is one of the main directions of developing high-strength IF steel at domestic and foreign plants [5,6]. Due to the good strengthening effect and low price of P element, the strength of IF steel can be improved by solution strengthening by adding appropriate P element and adjusting the proportion of Si and Mn element. The higher the plastic strain ratio r_90_ is, the better the stamping performance is. The research shows that the shape and size of inclusions, the grain size, and the proportion of favorable texture all affect the stamping performance of the strip [7,8]. Rare earth has become an important microalloy element in high value-added steel materials because of its large atomic size, unique outer electronic structure, variable valence state, and strong activity. The role of rare earth in steel can be summarized in the following aspects: Purification, modification, and microalloying [9,10]. The effect of rare earth Ce on the deep stamping property of high-strength IF steel containing phosphorus by industrial production and the application research of more than 50% yield is relatively less. In this paper, the influence of rare earth Ce on inclusions, grain size, and texture in the whole process of aluminum deoxidized IF steel casting and rolling was studied by an industrial test system, and the r_90_ value of a finished strip was characterized, which provides technical guidance for industrial application to improve the deep stamping property control of IF steel through new ways.

## 2. Test Method

### 2.1. Process

The experimental materials were produced in the Rare Earth Steel Plates Plant of Baotou Iron and Steel Group Corporation, located in Baotou, China. In the whole process of production, except adding rare earth, other processes were identical. The definition of the whole series of samples without adding rare earth is 1# and the whole series of samples with rare earth is 2#. The production process is described as follow.

**Figure 1:**
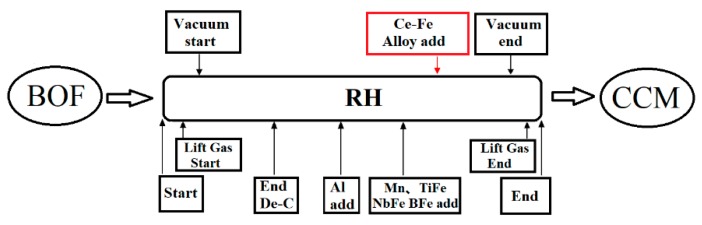
Ce-Fe alloy added during steelmaking processes.

(1) Smelting process. In the hot continuous rolling production line, the production contrast test of IF steel strengthened by phosphorus in the adjacent furnaces of the same pouring time was carried out, and the production process flow and rare earth addition time are shown in Figure 1.

During vacuum circulation degassing refining (RH) treatment, the limit vacuum degree was ≤0.106 Kpa, and the total vacuum treatment time was 30 min. Due to the strong oxidizability of rare earth, in order to avoid the oxygen oxidation brought on by other alloys during the alloying process, and to ensure the effective boron content in the steel, after aluminum deoxidization, the addition sequence of the alloy in the RH vacuum treatment process was ferromanganese, ferrotitanium, ferroniobium, and ferroboron. The 120 kg rare earth ferrocerium alloy (Ce content was 10%) was added at 3 min after alloying. After RH treatment, the sedation time was more than 15 min to ensure the inclusion would fully float. The recovery rate of rare earth Ce was 51%.

**Figure 2:**
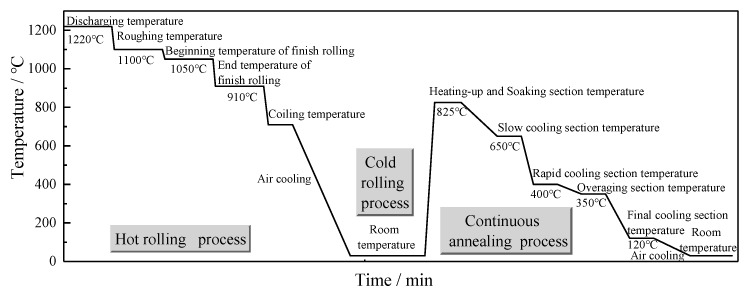
The specific technological process of each rolling process.

(2) Casting process. In the continuous casting (CC) process, strict protective casting measures against secondary oxidation were adopted to prevent nitrogen and oxygen increase. The chemical composition of the tundish sample was analyzed by direct reading spectrum analyzer (ARL-4460, Thermo Fisher Scientific, Switzerland), and the content of rare earth in the steel was detected by chemical analysis. The yield of Ce was 51%. Selected steel grades and the final chemical composition of the samples is shown in Table 1.

(3) Rolling processes. The specific technological process of each rolling process is shown in Figure 2.

### 2.2. Method

The size of the slab was 230 × 1550 mm^2^, and the samples of 1# and 2# were cut at the end of the second strand of the second slab of each heating, respectively. In order to compare and count the types and sizes of inclusions in the whole casting and rolling process and ensure the representativeness of the test data and the accuracy of the analysis, the sampling position was selected at 1/4 of the slab width and 1/4 of the slab thickness. The selected sample size was 10 × 10 × 10 mm.

The sampling positions of strips in hot rolling (HR), cold rolling (CR), and continuous annealing (CA) correspond to the slab positions, which were 1/4 of the width direction of the plate. The sample sizes were 4.3 × 10 × 10 mm^3^, 1.15 × 10 × 10 mm^3^, and 0.7 × 10 × 10 mm^3^.

The samples of CC and each rolling process were cut, inlaid, ground, and polished, and then the inclusions were detected by ASPEX-Scanning electron microscope (ASPEX-SEM, ASPEX eXplorer, ASPEX, Pembroke Park, FL, USA). In this study, the edge of the metallographic samples was avoided, and the field area of view was not less than 50 mm^2^. SEM (ZEISS LEO EVO 50HV, Carl Zeiss, Oberkochen, Germany) and energy dispersive spectrometer (EDS, EDAX, Mahwah, NJ, USA) were used to observe the morphology and composition of the typical inclusions found in the above samples.

The samples of each rolling process were etched by 4% nitric acid alcohol, the original morphology was extracted, and the three-dimensional morphology and composition of typical inclusions and second phase precipitates were observed by SEM-EDS.

The samples of the HR and CA processes were cut, inlaid, ground, and polished. After processing, the surface of the samples was etched with 4% nitric acid alcohol. Then, the microstructure of the samples was observed by optical microscope (Axio observer D1m, Carl Zeiss, Oberkochen, Germany) and the grain size were tested according to the standard GB/T 6394-2017.

The sample size of each rolling process was cut into 20 × 25 mm^2^, and then the sample was polished. The content and strength of favorable texture were analyzed by X-ray Diffraction (XRD, X’ Pert Pro MPD, Malvern Panalytical B.V., Almelo, The Netherlands).

The strip was sampled according to the standard GB/T 2975, rolling 20 coils in sample 1# and sample 2#, respectively. Each coil weighed 7 tons, and sampling was carried out at 1/4 of the width at 3 m from the tail of the strip. The mechanical properties of products were tested according to the standard GB/T 5027. Through the comparison of the above test results, the influence of rare earth addition on the deep stamping property of high-strength IF steel containing phosphorus was analyzed.

## 3. Results and Discussion

### 3.1. Effect of Rare Earth Ce on Inclusions

According to the statistics of the number, size, and proportion of all kinds of inclusions in the samples of casting and rolling process by ASPEX-SEM, the main types of inclusions in the samples 1# are Al_2_O_3_, Al-O-Ti, TiN, MnS, silicate, etc. The inclusions in the samples 2# are mainly Al-O-Ce, Ce-S-O, Ce-O, Al_2_O_3_, Al-O-Ti, TiN, MnS, etc.

**Figure 3:**
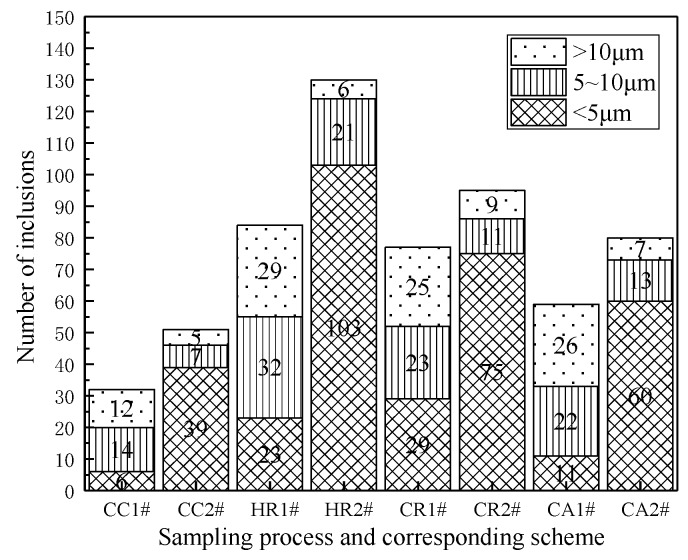
The statistics of inclusion size and quantity in each process.

Figure 3 shows the number of inclusions in different size ranges under corresponding processes and schemes. The amount of inclusion with CC, HR, CR, and CA process greater than 10 μm under the statistical field of view of sample 1# group were 12, 29, 25, 26, respectively. The sample 2# group were 5, 6, 9, and 7 respectively. The proportion of inclusions less than 5 μm in each process of sample 1# group were 18.7%, 27.4%, 37.7%, and 18.6%, respectively. The sample 2# group were 76.5%, 79.2%, 78.9%, and 75%, respectively. It can be seen that the inclusion scale decreased and the number of inclusions increased obviously in the whole casting and rolling process after adding rare earth.

Because of the high oxidation property of Ce, it will react with oxygen, sulfur, and other elements in the molten steel. The thermodynamic calculation of the rare earth compound formed by adding cerium was carried out.

**Figure 4:**
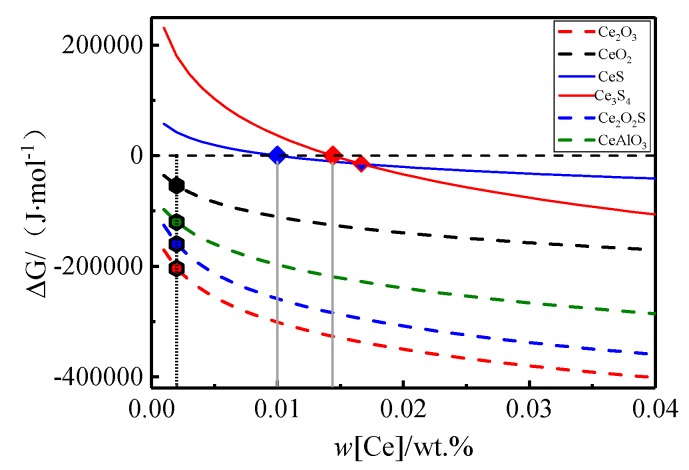
Precipitation of rare earth inclusions in steel.

Table 2 shows the chemical reactions related to the addition of rare earth to the molten steel and the standard Gibbs free energy [11,12]. According to the reaction and the activity coefficient of each element in the molten steel, the Gibbs free energy of production of each rare earth inclusion at 1873 K was calculated as shown in Figure 4. It can be concluded that when the content of wt.% Ce in the rare earth was less than 0.01, only CeAlO_3_, Ce_2_O_3_, Ce_2_O_2_S, and CeO_2_ were generated in the molten steel. When the content of wt.% Ce was greater than 0.01, CeS, Ce_3_S_4_, and other inclusions were successively precipitated out. As the alloying and solidification process of molten steel is unsteady, there may be different contents of inclusions, such as Al-O-Ce and S-O-Ce, in the steel [13].

**Figure 5:**
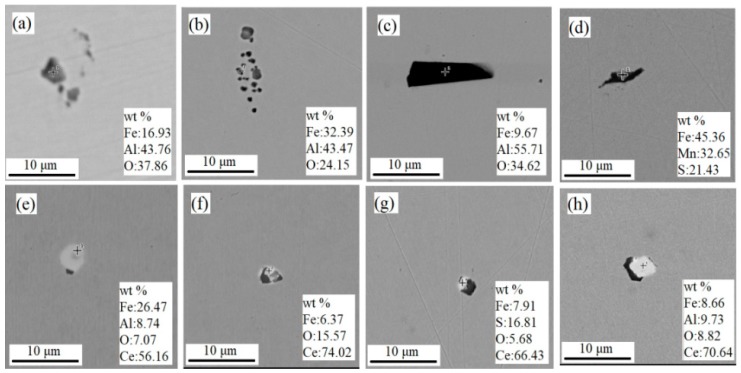
Comparison of typical inclusion morphology between the samples 1# and 2# in each process. 1#: (**a**) CC, (**b**) HR, (**c**) CR, (**d**) CA. 2#: (**e**) CC, (**f**) HR, (**g**) CR, (**h**) CA.

Figure 5 is a comparative analysis of the two-dimensional morphology of CC and each rolling process. It can be seen that the typical inclusions of sample 1# were mainly Al_2_O_3_ with sharp angle and long strip MnS with cluster shape, and the size can reach more than 10 μm. The inclusions in the samples 2# were mainly CeAlO_3_, Ce_2_O_3_, and Ce_2_O_2_S and the shape was round with a smooth surface. The size was 2–5 μm, and the distribution was independent dispersion.

Al_2_O_3_ inclusions are brittle inclusions with obvious edges and corners, and become chain-like distributions after rolling. Large size inclusions are easy to become the source point of steel fracture under stress, which is easy to scratch the matrix and produce the crack source [11]. Meanwhile, the long strip and large-size MnS inclusions in the aluminum deoxidized IF steel have a large amount of deformation in the deep stamping process, which is very easy to cause the impact of material transverse, radial toughness and plasticity, reduce the continuity of the structure, and cause the product stamping cracking and other problems [14].

When rare earth Ce was added to the steel, the combination of Ce with activity O and S in the steel had lower Gibbs free energy, and it was easy to generate CeAlO_3_, Ce_2_O_2_S, and Ce_2_O_3_. On the one hand, it reduced the concentration and supersaturation of aluminum and oxygen elements, and reduced the ability of single particle Al_2_O_3_ to aggregate into large-scale cluster inclusions [15]. On the other hand, Ce first combined with S in steel and precipitated earlier than MnS in the solidification process, resulting in small-size spherical inclusions, which can significantly reduce the size and quantity of MnS inclusions at various positions of the slab [16,17].

Therefore, Ce plays a role in the modification of inclusions. The morphology of inclusions changes from chain and strip to spherical. The size of the round inclusions was mostly about 2–5 μm. The distribution of the inclusions was dispersive, which is conducive to improving the structure continuity of the steel. The small spherical rare earth inclusions were not easy to deform in the process of impact deformation, which can slow down the stress concentration at the crack tip in the process of crack growth, thus playing an effect of cushioning and impeding crack growth [18]. In the process of hot working, the damage caused by large-scale inclusions and their shapes was reduced, and the anisotropy of materials was reduced. Besides, after the addition of rare earth, it preferentially segregated at the grain boundary, purified the grain boundary, and improved the grain boundary strength. The transition from intergranular fracture to transgranular fracture can effectively improve the impact toughness and deep stamping performance of the strip [19].

### 3.2. Effect of Rare Earth Ce on Microstructure Refinement of Steel Strip

**Figure 6:**
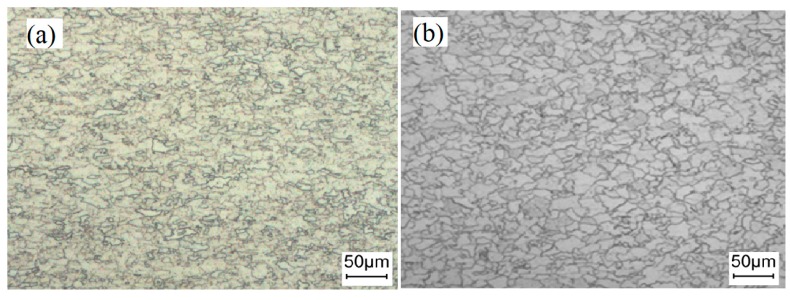

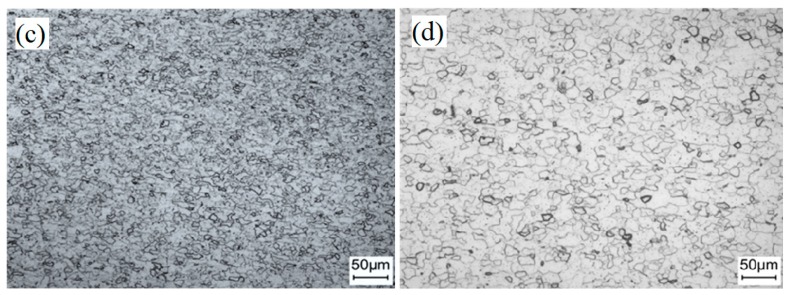
Comparison of microstructure of strip during rolling process. 1#: (**a**) HR, (**b**) CA. 2#: (**c**) HR, (**d**) CA.

Figure 6 is the comparison of microstructure and grain size detection of a strip in each rolling process. It can be seen that the microstructure was ferrite, the grain of sample 1# obviously had a certain orientation, grain growth was not enough, the structure was not fully recrystallized, some grains were isometric, and a small part of grains were long strip. The grain of sample 2# were more uniform than sample 1#. After annealing, the structure realized complete recrystallization, and the grains grew more evenly, all of which were transformed into equiaxed grains. After the test, the grain sizes of sample 1# were 7 and 9.5, and the sample 2# were 8.5 and 11. The results show that the structure of the strip was more uniform and compact, and the grain size was finer.

Due to the addition of rare earth Ce, a series of high melting point compounds such as CeO_2_, Ce_2_O_3_, Ce_2_O_2_S, and CeAlO_3_ with small size were formed in the steel. During the solidification process, homogeneous nucleation needed to generate a crystal nucleus larger than the critical size from the liquid phase, which required a high degree of supercooling. However, the activation energy of heterogeneous nucleation based on the second phase particles in the melt was greatly reduced. Based on the empirical electron theory and lattice mismatch theory [12,20], the lattice mismatch of CeO_2_, Ce_2_O_3_, Ce_2_O_2_S, and CeAlO_3_ was very small, which had a smaller interface free energy required for transformation. Its nucleation supercooling was much smaller than that of A1_2_O_3_, SiO_2_, and MnO, so it can increase the nucleation density and refine the grains.

In addition, based on the solidification theory, the difference between heterogeneous nucleation work and homogeneous nucleation work was 1/4[2-2cosθ-sin2θcosθ], the wetting tendency of crystal on inclusion surface was expressed by θ, and the wetting angle θ indicated the effectiveness of heterogeneous nucleation. The high surface activity of rare earth elements resulted in the decrease of interfacial tension, the increase of viscosity, the decrease of the wetting angle, and the production of smaller heterogeneous nucleation work [21]. The fine and dispersed rare earth compounds with high melting point were distributed in the steel. These particles can be used as heterogeneous nucleation points, effectively improving the nucleation rate, obtaining the inoculant effect for the heterogeneous nucleation of δ-Fe and γ-Fe, further expanding the equiaxed crystal area, limiting the development of columnar crystal, and thus realizing the refinement of solidification structure [22,23,24].

In the HR process, due to the large rare earth atoms, the solid solution in the crystal was limited, and the distortion energy caused by dissolution in the crystal was far greater than that dissolved in the grain boundary area, so the rare earth elements will preferentially segregate in the grain boundary [25,26]. However, the melting points of CeO_2_, Ce_2_O_3_, Ce_2_O_2_S, and CeAlO_3_ particles are relatively high, which can preferentially precipitate on the grain boundary, subgrain boundary, and dislocation line to hinder the grain boundary migration, stabilize the subgrain, slow down the process of dynamic recrystallization, static recrystallization, and grain growth, and obtain the rolled structure of grain refinement [20,27].

As a result of the heredity of the structure, based on the fine grains, the smaller parent phase austenite was produced in the CA process, which provides favorable conditions for the next refinement of ferrite structure (Figure 6). The finer and uniform grains indicated that more grains were involved in deformation, so that the strip had better plastic deformation. Meanwhile, the finer the grain was, the more the number of grains per unit volume was, the larger the total area of grain boundary was, the more dislocation barriers were generated, the more work was consumed before fracture, and the higher the plastic deformation resistance of metal was [18]. Therefore, the addition of rare earth can improve the grain size rating of the finished strip by 1.5 grades, which is conducive to improving the strength and plasticity of the strip.

### 3.3. Effect of Rare Earth Ce on Texture of Strip

The excellent deep stamping properties of IF steel mainly come from its {111} texture. Table 3 shows the statistics of the favorable texture content of the strip detected in each rolling process. It can be seen that the sample 2# percentage contents of the favorable texture {110} <001> in the HR process and {111} (< 110 > + < 112 >) in the CR and CA processes were 5.78%, 22.7%, and 24.16% respectively, which were greater than sample 1# (3.79%, 18.8%, and 21.62%) in the corresponding processes.

**Figure 7:**
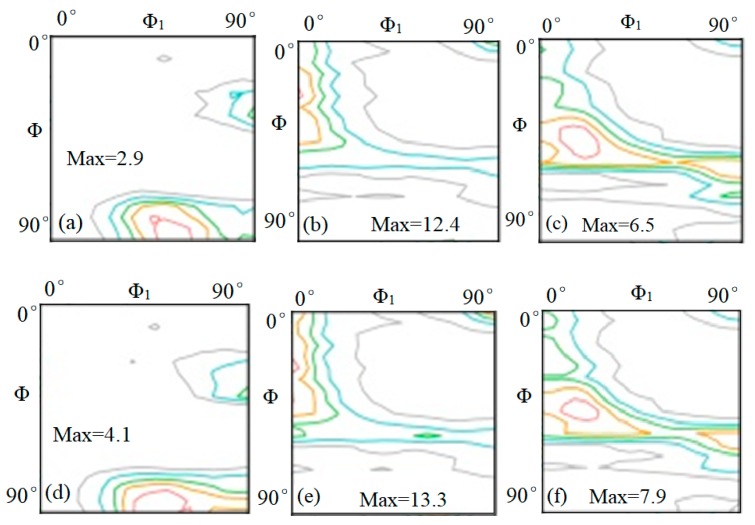
The ODF cross section (Ф_2_ = 45°) in the rolling process. 1#: (**a**) HR, (**b**) CR, (**c**) CA. 2#: (**d**) HR, (**e**) CR, (**f**) CA.

Figure 7 shows the content statistics of orientation distribution function (ODF) cross section (Ф_2_ = 45°) of strip steel detected in each rolling process. It can be seen that the sample2# polar density max values of texture in HR, CR, and CA processes were, respectively, 4.1, 13.3, and 7.9, which were greater than the sample1# (2.9, 12.4, and 6.5) in corresponding processes. The tensile strength of body centred cube lattice (BCC) metal in <111> direction was the highest, so the deformation resistance in <111> direction was the largest, and the <111> direction of {111} texture was perpendicular to the plate surface, so this texture made the steel plate difficult to deform in the thick direction during stamping, making the r value larger. The more the {111} texture content was, the higher the strength was and the higher the plastic strain ratio r value was [28,29]. Therefore, the sample 2# had better annealing structure and deep stamping properties.

**Figure 8:**
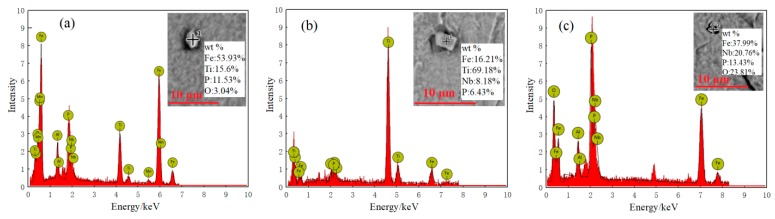
The Fe(Nb+Ti)P precipitation of sample 1# detected in the rolling process: (**a**) HR, (**b**) CR, (**c**) CA.

Figure 8 shows the typical Fe(Nb+Ti)P precipitates of the sample 1# observed by SEM in the rolling process. It can be seen from the figure that the types were FeTiP, Fe(Nb+Ti)P, FeNbP, etc., with the size range of 1–3 μm. No inclusion of this type was found in each field of view in the sample 2#. The strength of IF steel containing phosphorus was mainly enhanced by the solution strengthening effect of phosphorus. However, it was easy for phosphorus that precipitated in the precipitated phase of FeTiP to cause the grain boundary brittleness of steel due to grain boundary segregation. In the process of annealing and recrystallization, FeTiP played a role of a pinning effect on the grain boundary, hindered the growth of {111} oriented grains, and weakened the γ texture strength, which is harmful to the r value [30,31,32]. In addition, the precipitation of Fe(Nb+Ti)P will also consume solid solution P element, reduce the strength of the strip steel, consume Ti element, increase the interstitial atom C, N, and damage the formability of the material [33,34].

The results show that [35,36], because the formation process of Fe(Nb+Ti)P phase was that the mixed rich group of FeTi and FeNb phase formed first in steel, the Fe(Nb+Ti)P phase formed as P element diffused into the rich group of FeTi and FeNb. With the addition of rare earth, the diffusion ability of Nb and Ti elements dissolved in the steel was weakened, which led to a small number of FeTi and FeNb phases in the steel containing rare earth, and the precondition for the formation of Fe(Nb+Ti)P phase was restrained to some extent. Moreover, rare earth made the diffusion rate and ability of P element weaken. That is, the ability of P element diffusion into the phase of FeTi and FeNb was weakened, which made the further formation of Fe(Nb+Ti)P phase difficult.

Other studies show that [26] the atomic radius of Ce was about 50% larger than that of Fe, which is a solid solution in the matrix, resulting in a large lattice distortion energy and an increase in the system energy. Because the interaction between Ce and dislocation was larger than that of P, according to the theory of solute atom equilibrium segregation, Ce and P had competitive segregation, and Ce interacted with dislocation first, occupying the distortion area and vacancy on the grain boundary preferentially, thus inhibiting the segregation of P on the grain boundary and hindering the precipitation of Fe(Nb+Ti)P phase. Therefore, Ce can inhibit the precipitation of P element and Fe(Nb+Ti)P phase, effectively increasing the solid solution amount of P element in steel, improving the solid solution strengthening effect of P element in high-strength IF steel, and obtaining a large proportion of {111} favorable texture, which is conducive to improving the formability index r_90_ value.

### 3.4. Comparison of Stamping Properties

**Figure 9:**
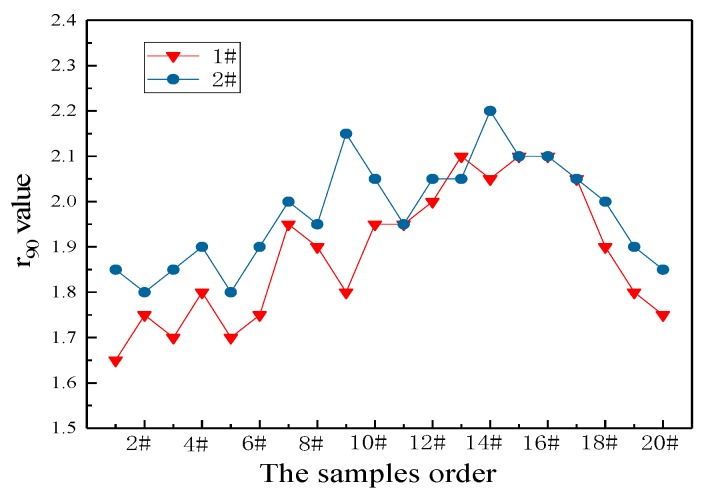
Comparison of r_90_ values.

Based on the above analysis, according to the comparison results of the mechanical properties of the samples in Figure 9, the overall r_90_ value of the sample 2# was higher than that of the sample 1#. The high-strength IF steel containing phosphorus with added rare earth had a better deep drawing property. After rare earth addition, the inclusion size was reduced, the inclusion morphology was optimized, the solidification nucleation rate was increased, the as-cast and as-rolled grains were refined, and the strength and toughness of the strip were effectively improved by fine grain strengthening. Meanwhile, the solution strengthening effect of P element in high-strength IF steel was improved, a large proportion of {111} favorable texture was obtained, and the r_90_ value of the product was increased. The above results have certain guiding significance for improving the deep stamping property of high-strength IF steel and lightweight steel application.

## 4. Conclusions

(1) Based on the addition of rare earth in steel, the combination of Ce with activity O and S in the steel had lower Gibbs free energy, and it was easy to generate CeAlO_3_, Ce_2_O_2_S, and Ce_2_O_3_. Meanwhile, the inclusion size decreased obviously, but the amount of inclusions increased. The morphology of inclusions changed from chain and strip to spherical. The size of rare earth inclusions was mostly about 2–5 μm, distributed and dispersed, and their elastic modulus was close to that of steel matrix, which is conducive to improving the structure continuity of steel.
(2) The rare earth compound had a high melting point. As a heterogeneous nucleation point, the nucleation rate was increased and the solidification structure was refined. Meanwhile, in the process of rolling heat treatment, rare earth can preferentially segregate grain boundaries to slow down grain growth. The addition of rare earth can improve the grain size rating of a continuous annealing strip by 1.5 grades, which is conducive to improving the strength and plasticity of strip.
(3) Rare earth Ce can inhibit the segregation of P element at the grain boundary and the precipitation of Fe(Nb+Ti)P phase. It can effectively increase the solid solution amount of P element in steel, improve the solid solution strengthening effect of P element in high-strength IF steel, and obtain a large proportion of {111} favorable texture, which is conducive to improving the stamping formability index r_90_ value.

## Figures and Tables

**Table 1:** Chemical composition of the samples (wt.%).

Heat Number	C	Si	Mn	P	S	Als ^1^	Nb	Ti	B	Ce
2nd	0.0020	0.15	0.68	0.078	0.005	0.029	0.027	0.022	0.0012
3nd	0.0018	0.14	0.70	0.074	0.005	0.032	0.025	0.025	0.0010	0.0022

^1^ Al_s_, acid soluble aluminum.

**Table 2:** Thermodynamic calculation of the rare earth inclusions formation.

Reaction Equations in Molten Steel	$\Delta {G^{\theta }=A+B\;\times \;T,\;J\cdot \mathrm{mol}}^{-1}$	
	A	B
2Al + 3O = Al_2_O_3_ (s)	−1,208,271	390.91
2Ce + 3O = Ce_2_O_3_ (s)	−1,431,090	360.06
Ce + 2O = CeO_2_ (s)	−854,274.7	249.11
Ce + S = CeS (s)	−422,783	120.58
3Ce + 4S = Ce_3_S_4_ (s)	−1,493,010	438.9
2Ce + 2O + S = Ce_2_O_2_S (s)	−1,353,592.4	331.6
Ce + Al + 3O = CeAlO_3_ (s)	−1,366,460	364

**Table 3:** The statistics of favorable texture content in each rolling process.

Processes	HR {110} <001> /%	CR {111} (<110> + <112>) /%	CA {111} (<110> + <112> /%)
1#	3.79	18.8	21.62
2#	5.78	22.7	24.16
